# Effect of optical correction on subfoveal choroidal thickness in children with anisohypermetropic amblyopia

**DOI:** 10.1371/journal.pone.0189735

**Published:** 2017-12-19

**Authors:** Tomo Nishi, Tetsuo Ueda, Yuutaro Mizusawa, Kentaro Semba, Kayo Shinomiya, Yoshinori Mitamura, Taiji Sakamoto, Nahoko Ogata

**Affiliations:** 1 Department of Ophthalmology, Nara Medical University, Nara, Japan; 2 Department of Ophthalmology, Institute of Biomedical Sciences, Tokushima University Graduate School, Tokushima, Japan; 3 Department of Ophthalmology, Kagoshima University Graduate School of Medical and Dental Sciences, Kagoshima, Japan; Massachusetts Eye & Ear Infirmary, Harvard Medical School, UNITED STATES

## Abstract

The purpose of this study was to determine the effect of optical correction on the best-corrected visual acuity (BCVA) and subfoveal choroidal thickness (CT) in the eyes of children with anisohypermetropic amblyopia. Twenty-four anisohypermetropic amblyopic eyes and their fellow eyes of 24 patients and twenty-three eyes of 23 age-matched control children were studied. After one year of optical correction, the BCVA in the anisohypermetropic amblyopic eyes was significantly improved. Before the treatment, the mean subfoveal CT in the amblyopic eyes was 351.9 ± 59.4 μm which was significantly thicker than that of control eyes at 302.4 ± 63.2 μm. After the treatment, the amount of change in the subfoveal CT in the amblyopic and fellow eyes was greater than that in the control eyes. The amblyopic and fellow eyes with thicker choroids had a greater thinning of the choroid whereas eyes with thinner choroids had a greater thickening of the choroid. We conclude that wearing corrective lenses improves the visual acuity, and induces changes of the subfoveal CT in eyes with anisohypermetropic amblyopia.

## Introduction

Anisohypermetropic amblyopia is a developmental disorder of the visual system that is characterized by reduced visual acuity in the absence of visible ocular pathological changes. [1] Experimental evidence has shown functional changes in the brain of animal models of amblyopia without anatomical changes. [2–4] Thus, amblyopia is believed to be caused by a deprivation of clear vision during the period of neural plasticity early in life. [5,6] We recently found that eyes of children with anisohypermetropic amblyopic have differences in the retinal microstructures and choroidal structures of the fovea from that of normal eyes. [7–9]

The choroid plays an important role in the modulation of the refractive state, and its development can be affected by refractive errors.[10–13] There have been several studies on the choroidal thickness (CT) in children. [14–18] It was reported that the subfoveal choroid was significantly thicker in anisohypermetropic amblyopic eyes than in the fellow eyes and control eyes. [8]

Many clinical studies have shown that the best-corrected visual acuity (BCVA) is significantly improved in amblyopic eyes after wearing corrective lenses. [19–22] Furthermore, an increase in the outer segment length of the fovea was detected in the amblyopic eyes after optical treatments. [7] However, it has not been determined whether wearing optical correction for anisohypermetropia will alter the CT in the eyes of children with amblyopia. [8,14–18]

Thus, the purpose of this study was to determine the effects of wearing corrective optical lenses on the BCVA and CT in children with anisohypermetropic amblyopia. To accomplish this, the BCVA and the CT were measured before and after wearing the full optical correction for one year.

## Methods

### Patients and controls

This was a retrospective, comparative, observational study conducted from April 2012 to February 2016 at Nara Medical University Hospital and Tokushima University Hospital. The protocol of this study conformed to the tenets of the Declaration of Helsinki and was approved by the Internal Review Board of the Nara Medical University and Tokushima University. An informed consent was obtained from all patients and parents to perform the measurements and to review the medical records.

The medical charts of amblyopic patients whose follow-up period was at least one year were retrospectively studied. An eye was diagnosed as being amblyopic when the BCVA was worse than 20/30 in one eye, and the it was at least two Snellen lines worse than that of the fellow eye. Anisohypermetropia was defined as a difference in refractive power (spherical equivalent) of 1.5 D or more between the two eyes. [21] The control group was composed of children whose BCVA was ≥20/20, whose age was from 3 to 7 years, whose refractive error was -1.5 to +1.5 diopters, and who had no ocular disorders in both eyes. We examined the control children at the Nara Medical University Hospital and at the nursery school of Tokushima University Hospital during a group medical examination. We selected the right eye of the control eyes. Patients with organic eye diseases, strabismus, history of intraocular surgery, laser treatment, cataract, glaucoma, or any retinal disorders were excluded. In addition, patients who could not cooperate with the optical coherence tomography (OCT) examinations were also excluded.

Twenty-four amblyopic eyes and their fellow eyes of 24 patients with anisohypermetropic amblyopia (6.0 ± 1.8 years, mean ± standard deviation, range 3 to 9 years) and twenty-three eyes of 23 control children (5.1 ± 1.1 years, range 3 to 7 years) were studied. The amblyopic patients included 10 boys and 14 girls. The control children included 10 boys and 13 girls. The choroidal thickness, axial length, and visual acuity of nineteen of these 24 amblyopic patients and ten controls before treatment have been reported. [7]

All participants had a comprehensive eye examination including measurements of the best-corrected visual acuity (BCVA), slit-lamp examinations, extraocular motility assessments, dilated ophthalmoscopy, and OCT recordings. The visual acuity was measured with a standard Snellen chart, and the decimal visual acuity was converted to the logarithm of the minimal angle of resolution (logMAR) units for the statistical analyses. The refractive errors (spherical equivalents) were measured with an auto refracto/keratometer (KR-8100, RM8900, Topcon, Tokyo, Japan) after a combination of topical 1% cyclopentolate and 2.5% phenylephrine. The axial length of the eye was measured with the A-mode ultrasound (IOL Master, Carl Zeiss Meditec, Dublin, CA,or the AL-2000 TOMEY, Nagoya, Japan). All examinations were performed between 13:00 to 15:00 hours to avoid diurnal variations. [23]

The subfoveal CT of all patients was measured from the images obtained by spectral-domain optical coherence tomography (SD-OCT) with the enhanced depth imaging program as reported. [7] Briefly, the subfoveal CT was measured manually as the distance between the basal edge of the retinal pigment epithelium (RPE) and the chorioscleral border. Two observers who were masked as to whether the eye was amblyopic selected the best scan and measured the CT independently. The final thickness was calculated as the arithmetic mean of the measurements of the two observers. The inter-observer reproducibility was evaluated using intraclass correlation coefficients (ICCs). The changes of the subfoveal CT were compared to the baseline subfoveal chorodial thickness. The percentage change in the subfoveal CT was calculated as the change of the subfoveal CT divided by the baseline subfoveal CT.

### Optical treatments

This was an observational study in which all patients received standard of care treatment. Spectacles for the amblyopic eyes and fellow eyes were prescribed at the first visit, and the power of the spectacles was the full correction of the refractive errors which was determined by an auto refracto/keratometer after cycloplegia by a combination of topical 1% cyclopentolate and 2.5% phenylephrine. All patients underwent one year of optical treatment but patching therapy was added after 12 weeks if needed in 15 cases. The patching treatment consisted of an adhesive patch worn over the fixing eye for 2–3 hours/day.

One year after beginning of the optical treatment, a complete examination was performed, and the effects of the optical correction and patching on the anisohypermetropic amblyopic eyes were evaluated by the changes in the BCVA, axial length, CT, and OCT. All examinations were done in a masked fashion. The ocular examinations of the control children were conducted at the baseline and after one year without optical treatment.

### Statistical analyses

The data are expressed as the means ± standard deviations (SDs). The age, BCVA, axial length, and refractive error (spherical equivalent) of the amblyopic, the fellow eyes, and the control eyes were compared by one-way ANOVA with post-hoc Tukey tests. The significance of the correlations between the baseline subfoveal CT and the rate of changes in the subfoveal CT was determined by Pearson`s correlation coefficient.

$$The\;rate\;of\;changes\;in\;the\;subfoveal\;CT=\frac{The\;subfoveal\;CT\;after\;one\;year-Baseline\;subfoveal\;CT}{Baseline\;subfoveal\;CT}\times \;100\;\left(\%\right)$$ (1)

Statistical analyses were performed using a statistical software (SPSS version 21.0; SPSS Inc., Chicago, IL).

## Results

### Demographic data before optical correction

The mean BCVA before the optical correction was 0.33 ± 0.16 logMAR units in the amblyopic eyes, -0.02 ± 0.07 logMAR units in the fellow eyes, and -0.02 ± 0.04 logMAR units in the control eyes (Table 1). The mean refractive error was +4.35 ± 2.02 diopters (D) in the amblyopic eyes, +2.00 ± 1.73 D in the fellow eyes, and +0.28 ± 0.69 D in the control eyes (Table 1). The mean axial length of the amblyopic eyes before the treatment was 21.27 ± 0.74 mm, that of fellow eyes was 21.97 ± 0.84 mm, and that of control eyes was 21.73 ± 0.69 mm (Table 1).

**Table 1:** Demographics of amblyopic patients and controls at the baseline.

	Amblyopic eyes (n = 24)	Fellow eyes (n = 24)	Control eyes (n = 23)	P^1^ Amblyopia vs Fellow vs Control	P^2^ Amblyopia vs Fellow	P^2^ Amblyopia vs Control	P^2^ Fellow vs Control
**Visual Acuity (logMAR)**	0.33±0.16	-0.02±0.07	-0.02±0.04	<0.001	<0.001	<0.001	0.98
**Refractive error (Spherical equivalent) (D)**	+4.35±2.02	+2.00±1.73	+0.28±0.69	<0.001	0.001	<0.001	<0.001
**Axial length (mm)**	21.27±0.74	21.97±0.84	21.73±0.69	0.008	0.005	0.36	0.20
**Subfoveal CT (μm)**	351.9±59.4	297.7±63.6	302.4±63.2	0.006	0.01	0.02	0.96

^1.^ one way ANOVA

^2.^ Tukey test

Data are expressed as the means ± standard deviations

### Changes of visual acuity, refractive error, axial length, and CT

One year after the wearing the full correction, the BCVA was significantly improved from 0.33 ± 0.16 logMAR units to 0.05 ± 0.13 logMAR units in the amblyopic eyes (*P* = 0.001; paired *t* test). However, the refractive error did not change significantly after wearing the optical correction in the amblyopic eyes and the fellow eyes (Table 2).

**Table 2:** Demographics of amblyopic patients and controls after one year.

	Amblyopic eyes (n = 24)	Fellow eyes (n = 24)	Control eyes (n = 23)	P^1^ Amblyopia vs Fellow vs Control	P^2^ Amblyopia vs Fellow	P^2^ Amblyopia vs Control	P^2^ Fellow vs Control
**Visual Acuity (logMAR)**	0.05±0.13	-0.01±0.03	-0.05±0.05	<0.001	0.03	<0.001	0.24
**Refractive error (Spherical equivalent) (D)**	+4.33±2.00	+1.89±1.47	+0.15±0.94	<0.001	0.001	<0.001	0.01
**Axial length (mm)**	21.49±0.81	22.00±0.89	22.18±0.55	0.04	0.08	0.06	0.95
**Subfoveal CT (μm)**	346.0±59.8	303.1±62.5	294.5±63.3	0.01	0.05	0.02	0.9

^1.^ one way ANOVA

^2.^ Tukey test

Data are expressed as means ± standard deviations

Before wearing the optical correction, the mean subfoveal CT in the amblyopic eyes was 351.9 ± 59.4 μm which was significantly thicker than that of fellow eyes (297.7 ± 63.6 μm; *P* = 0.01, Tukey test) and that of the control eyes (302.4 ± 63.2 μm; *P* = 0.02, Tukey test; Table 1). The inter-observer reproducibility was excellent (ICC = 0.77).

The mean subfoveal CT of the amblyopic eyes was 346.0 ± 59.8 μm after one year of wearing the optical correction, and it was still significantly thicker than that of control eyes at 294.5 ± 63.3 μm, *P* = 0.02; Tukey test. However, the difference between the amblyopic eyes and the fellow eyes was not significant (Table 2).

In the amblyopic and fellow eyes wearing plus lenses, the subfoveal CT increased in 24 of 48 eyes and decreased in 22 of 48 eyes, and showed no change in 2 of 48 eyes. The CT in the control eyes increased in 8 of 23 eyes, decreased in 14 of 23 eyes, and showed no change in 1 of 23 eyes after one year.

**Fig 1:**
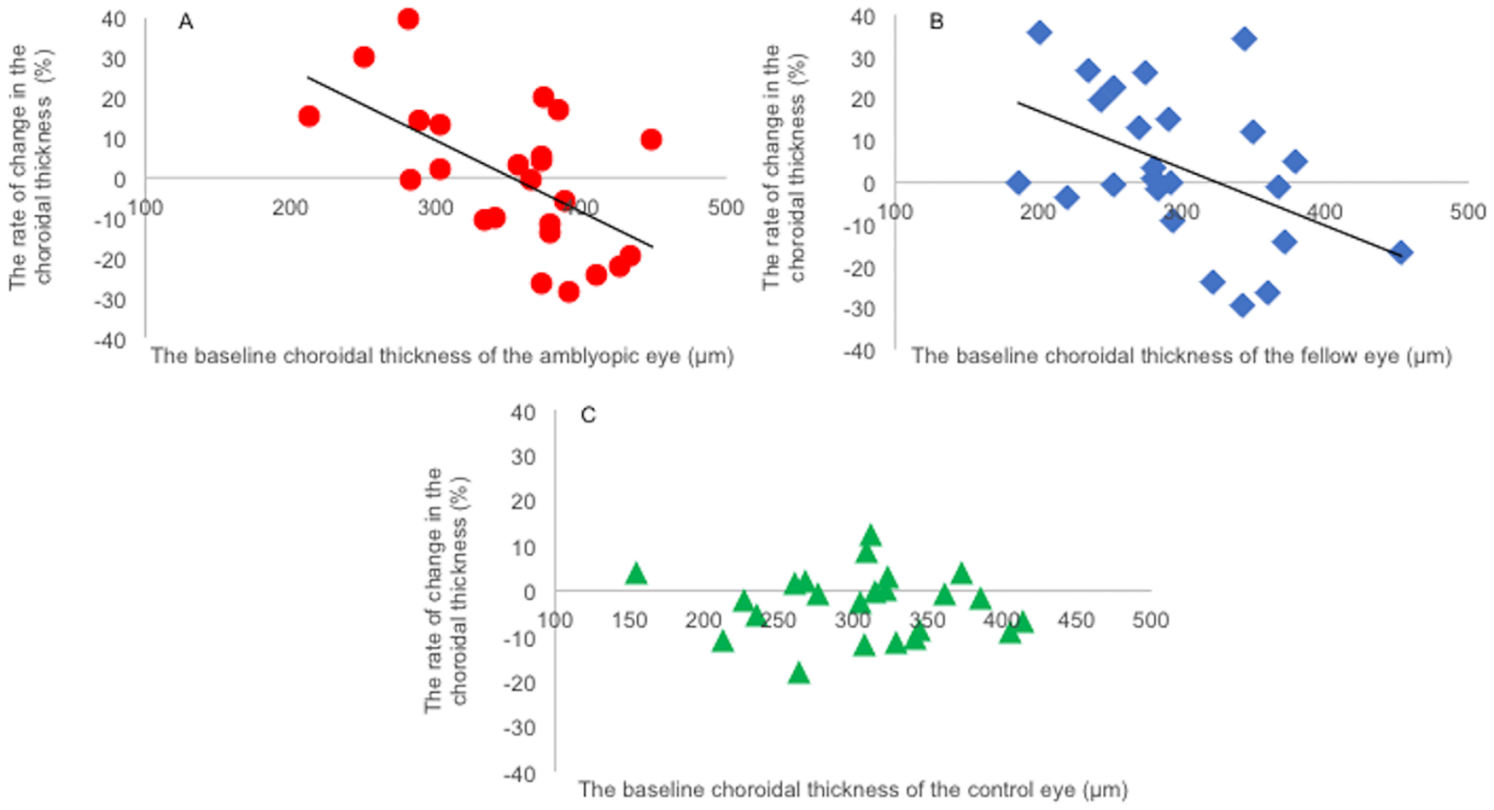
Rate of change in subfoveal choroidal thickness (CT). A: Changes of the subfoveal CT of amblyopic eyes in patients with anisohypermetropic amblyopia. The thicker choroids became thinner and thinner choroids became thicker. There was a negative correlation between the rate of change in the subfoveal choroidal thickness and the baseline subfoveal choroidal thickness. (*r* = -0.59, *P* = 0.003; Pearson’s correlation coefficient). B: Changes of the subfoveal CT of the fellow eyes in patients with anisohypermetropic amblyopia. The thicker choroid became thinner and thinner choroid became thicker. There was a negative correlation between the rate of change in the subfoveal choroidal thickness and the baseline subfoveal choroidal thickness. (*r* = -0.48, *P* = 0.02; Pearson’s correlation coefficient). C: Changes of the subfoveal CT of control eyes. There was no correlation between the rate of change in the subfoveal choroidal thickness and the baseline subfoveal choroidal thickness. (*r* = -0.15, *P* = 0.49; Pearson’s correlation coefficient).

Interestingly, the subfoveal CT in eyes with thicker choroid tended to decrease and the eyes with thinner choroid tended to increase in both the amblyopic and fellow eyes. There was a negative correlation between the rate of the changes in the subfoveal CT and the baseline subfoveal CT in the amblyopic and fellow eyes (amblyopic eyes: *r* = -0.59, *P* = 0.003; Pearson’s correlation coefficient; Fig 1A, fellow eyes: *r* = -0.48, *P* = 0.02; Pearson’s correlation coefficient; Fig 1B). There was no significant changes in the control eyes (*r* = -0.15, *P* = 0.49; Pearson’s correlation coefficient; Fig 1C).

### Two representative anisohypermetropic amblyopic patients and one control subject

**Fig 2:**
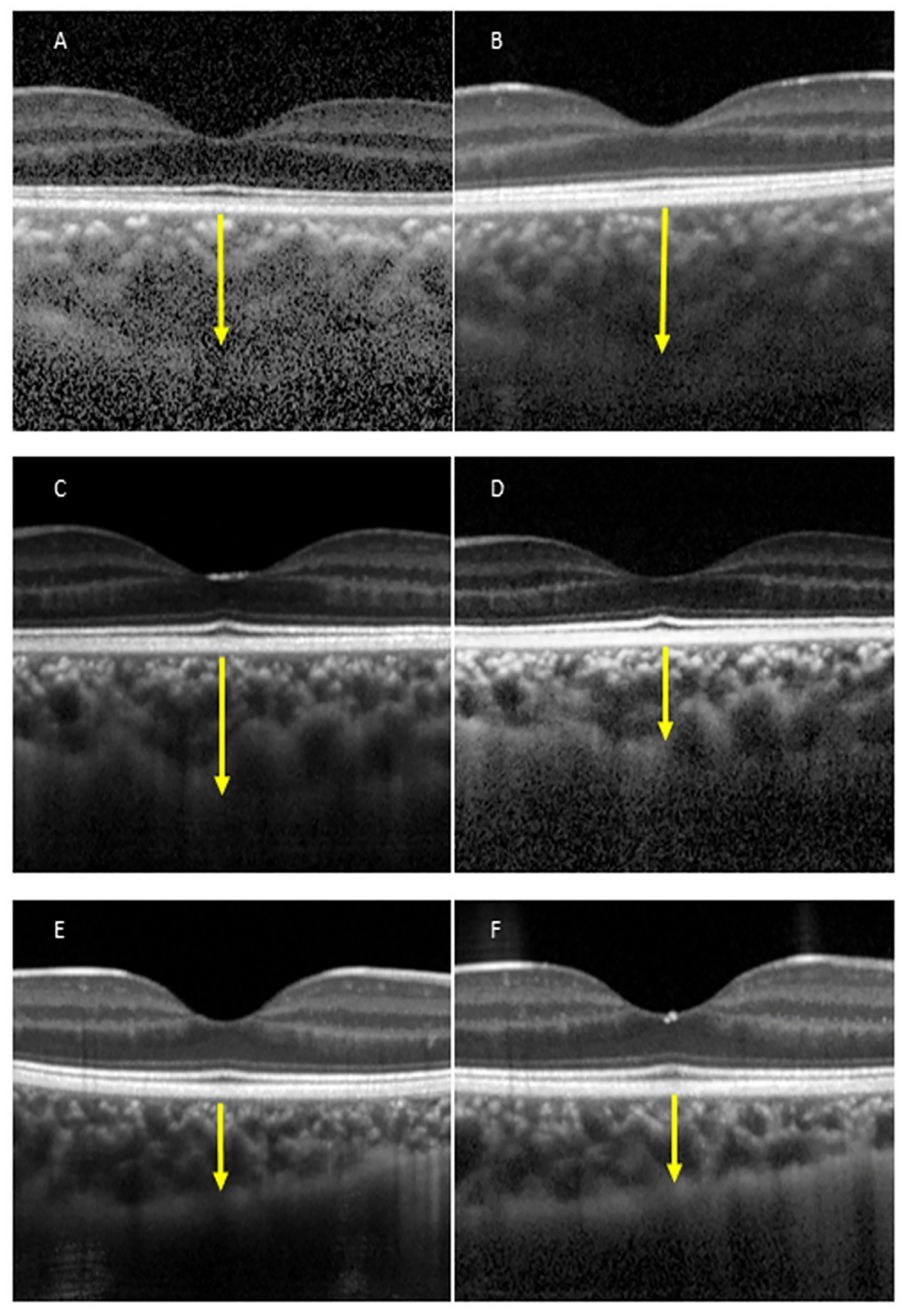
Enhanced depth imaging spectral domain optical coherence tomographic (EDI-SD-OCT) images of an anisohypermetropic amblyopic eye and control eye. A: EDI-SD-OCT image of the amblyopic eye at the baseline. B: EDI-SD-OCT image of the amblyopic eye at one year after the full optical correction treatment. C: EDI-SD-OCT image of an amblyopic eye at the baseline. D: EDI-SD-OCT image of an amblyopic eye at one year after the treatment. E: EDI-SD-OCT image of a control eye at the baseline. F: EDI-SD-OCT image of a control eye after one year. Arrow indicates the subfoveal CT. All images are horizontal scan.

The findings in a representative 4-year-old amblyopic patient whose CT was increased after wearing the full correction are shown in Fig 2A and 2B. Our initial examination showed that his BCVA was 0.5 logMAR units in the left amblyopic eye. One year after wearing the optical correction, the BCVA of the amblyopic eye improved to 0 logMAR units. At the baseline, the subfoveal CT in the amblyopic eye (left eye) was 281 μm (Fig 2A), and one year after the treatment, the thickness of the subfoveal choroid in the amblyopic eye was 393 μm (Fig 2B) which was thicker than the baseline.

The findings in a representative 9-year-old amblyopic patient whose CT was decreased are shown in Fig 2C and 2D. Our initial examination showed that his BCVA was 0.5 logMAR units in the left amblyopic eye which improved to 0.2 logMAR units one year after beginning the treatment. At the baseline, the subfoveal CT in the amblyopic eye (left eye) was 391 μm (Fig 2C), and one year after the treatment, the thickness of the subfoveal choroid in the amblyopic eye was 279 μm (Fig 2D) which was thinner than that at the baseline.

The findings in a 5-year old control child are shown in Fig 2E and 2F. At the baseline, the subfoveal CT in the control eye (right eye) was 235 μm (Fig 2E). One year after the baseline, the thickness of the subfoveal choroid in the control eye was 222 μm (Fig 2F) which was not significantly different from that at the baseline. The arrows indicate the subfoveal choroidal thickness. All images are horizontal scans.

## Discussion

Our results showed that wearing optical corrective spectacle lenses on the amblyopic eyes improved the visual acuity significantly although the refractive error did not change after one year. The changes in the subfoveal CT in the amblyopic and fellow eyes were greater than that of the control eyes.

We recently presented the foveal microstructural changes after optical treatments in eyes of children with anisohypermetropic amblyopia. [7] However, a consensus on whether the changes of the choroidal ultrastructure after optical treatment in amblyopic eyes has not been reached. We have reported that the subfoveal CT before the treatment in amblyopic eyes was thicker than that of the fellow and control eyes. [8] Our results in this study showed that the subfoveal CT of amblyopic eyes was still thicker than that of the control eyes one year after the treatment. However, the change of the subfoveal CT in amblyopic eyes and fellow eyes was significantly greater than that of control eyes.

Eyes with a thicker choroid had choroidal thinning and eyes with thinner choroid had choroidal thickening both in the amblyopic and the fellow eyes after wearing the corrective lenses. Thus, wearing full corrective lenses improves the visual acuity, and induces compensatory changes of the subfoveal CT in anisohypermetropic amblyopic eyes. Therefore, the differences in the subfoveal CT between amblyopic, fellow, and control eyes became smaller. It appears that the choroid in amblyopic eyes underwent emmetropization without changes in the axial length. Optical treatment induced the changes of not only the retinal thickness[7] but also the CT.

The choroid has been shown to play an important role in emmetropization and refractive error development in young animals. [24,25] Hyperopic defocus by negative lenses induced choroidal thinning and accelerated the growth of the axial length in the eyes of chicks and monkeys. [24,25] On the other hand, myopic defocus by plus lenses promoted choroidal thickening and slowing of the elongation of the axial length. [24,25]

The choroidal thickness of the eyes is different at different ages, races, and study samples. In a Chinese study of 6026 children ages 6 to 18 years, the mean subfoveal choroidal thickness was 283 μm [26] while in an Australian study of 194 children ages 4 to 12 years, the mean subfoveal choroidal thickness was 330 μm. [27] In our study, the mean subfoveal choroidal thickness was 302 μm which is somewhat comparable to the values in these previous studies. [25,26] A cross sectional study [19] of primarily emmetropic children found that the age-related increase in the CT was approximately 9 μm/year.

There are limitations in this study. Only a small number of anisohypermetropic amblyopic eyes of Japanese children was investigated, thus further studies including a larger number of subjects will be necessary to confirm our findings. In addition, we measured the changes after one year of full optical correction, thus a longer follow-up is necessary.

In conclusion, wearing corrective plus lenses by amblyopic eyes improves the visual acuity significantly after one year. The changes of the subfoveal CT in amblyopic and fellow eyes was greater than that in control eyes. In addition, the amblyopic eyes and fellow eyes with thicker choroids had more choroidal thinning and the eye with thinner choroids had more choroidal thickening. Thus, optical correction promoted compensatory changes of the subfoveal CT in amblyopic eyes.
